# New Appliances and Things Medical

**Published:** 1903-10-24

**Authors:** 


### NEW APPLIANCES AND THINGS MEDICAL.

[We shall be glad to receive at our Office, 28 & 29 Southampton Street, Strand, London, W.C., from the manufacturers, specimens of all new preparations and appliances which may be brought out from time to time.]

### MILNE'S SURGICAL DRESSINGS AND APPLIANCES.

(The Galen Manufacturing Company, Limited, Wilson Street, New Cross Road, London, E.C.)

Mr. John Milne was associated with Lord (then Dr.) Lister in the earliest experiments on the action of antiseptics in Edinburgh, and came to London with that distinguished surgeon, to carry on the manufacture of surgical dressings according to his exact instructions. This fact alone, is sufficient to guarantee the trustworthiness of these appliances, and we find this firm's cyanide gauze, boracic lint, and alembroth wool and other dressings, everything that is excellent. A sterilised ribbon gauze is introduced for the plugging of wounds and cavities; being very soft and having a woven edge to prevent fraying, it seems particularly suitable for this purpose. Milne's "Battist," a waterproof preparation to be used instead of oil-silk, has many recommendations. It is grease and spirit-proof, can be boiled for two hours without injury and withstands all antiseptics. The washable tensile elastic bandage without indiarubber is highly to be commended; it is extremely light, cool, and porous, and very superior to the ordinary elastic bandage used for support. Milne's aseptic ligatures, attached to a rigid frame in a glass tube with indiarubber cork, have the advantage of necessitating no waste, no handling of the ligature, and each length comes out in a straight thread, instead of the curl met with when reels are used. A useful vaccination pad, and an antiseptic soap containing beta-naphthol, laurel camphor and cymene, specially recommended for use after operations, are other first-class appliances manufactured by this firm.

### LYGOSIN-SODIUM (ZIMMER'S).

(London Agents: Widenmann, Broicher and Co., 33 Lime Street, London, E.C.)

This new preparation has been largely experimented with on the continent, for the treatment of uterine gonorrhœa. It is somewhat doubtful how often, and to what extent, female gonorrhœa involves the uterus, but it is highly probable from recent observations that it more often does so than was originally believed, existing there perhaps for years, retaining its infectiousness and gradually setting up complications of a serious nature. The treatment of uterine gonorrhœa has not up till now been as satisfactory as it might have been, so that we must welcome "sodium lygosinate," which seems experimentally and clinically to be an effective anti-gonorrhœic. In strengths of 1 in 3,000 to 1 in 1,500, it is injected into the uterine cavity with most excellent results, where it causes no irritation to speak of or damage to the tissues. It appears, therefore, that in this preparation, we have a valuable remedy.

### PEPSALIA.

(Cerebos, Limited, Newcastle-on-Tyne.)

This preparation is described as Nature's Digestive. It consists of table-salt with a small percentage of pepsin added. Though the use of artificial aids to digestion must be carefully guarded against abuse, yet to those who have to have recourse to such, "Pepsalia" is most handy and useful.

### EUCRYL PREPARATIONS.

(Mayor & Co, Limited, Hull)

These preparations, containing eucalyptus, pine oil, and coal tar derivatives as their disinfectant bases, are extremely pleasant to use. Bath Eucryl is particularly agreeable and adds greatly to the enjoyment of a bath, having also a somewhat stimulating action. The Eucryl fluid disinfectant forms an opalescent solution with water, and leaves a most desirable odour on the hands, an acceptable quality to medical men. A toilet soap, ointment, and tooth-powder with Eucryl as their bases are also to be obtained. All these preparations seem to be efficacious and in every way excellent.

